# Development of Electromagnetic Acoustic Transducer System for Coin Classification

**DOI:** 10.3390/s22239055

**Published:** 2022-11-22

**Authors:** Duy-Vinh Dao, Jen-Tzong Jeng, Van-Dong Doan, Huu-Thang Nguyen, Bo-Yao Liang

**Affiliations:** Department of Mechanical Engineering, National Kaohsiung University of Science and Technology, Kaohsiung 807618, Taiwan

**Keywords:** electromagnetic acoustic transducer, coin classification, non-destructive testing

## Abstract

In this work, a method for identifying counterfeit coins based on an electromagnetic acoustic transducer (EMAT) to detect the difference in the coin’s natural acoustic frequency response is presented. In the experimental system, the acoustic oscillation induced by a pulsed magnetic field is received by a microphone and recorded by an oscilloscope. The natural acoustic frequency of the coin is resolved by the fast Fourier transform (FFT) method on the computer. It is found that the natural frequencies of the possible counterfeit coins deviate significantly from the standard ranges of 16.9 to 17.4 kHz for the authentic 50 New Taiwan Dollar (NTD) coins. The observed natural frequencies of the coin are consistent with the values predicted by analytical estimation. We also built a prototype EMAT coin classification system to detect the natural acoustic frequency by direct frequency counting using a microcontroller. The prototype system demonstrates that a counterfeit coin can be identified by its natural frequency in less than 30 ms using the EMAT method. The proposed technique can be applied to the vending machine to improve the accuracy in discriminating between authentic and counterfeit coins.

## 1. Introduction

Nowadays, the coins are not only used for commodity exchange, but may also carry information about the language, religion, sovereignty, and national heroes of the country. They are essential in daily life and are frequently used in vending machines, ticket machines, game machines, and payphones. However, counterfeits of higher-value coins frequently exist. The existence of fake coins often undermines the local economy and even results in the legal liability of innocent consumers. It is necessary to develop a reliable technology to identify counterfeit coins to avoid possible economic loss. In Taiwan, the 50 New Taiwan Dollar (NTD) coin is a widely used high-value coin (equivalent to about two US dollars), and it is also the target of the counterfeiter [1]. Although the mark, texture, and weight of the counterfeit coins were similar to those of real ones, most of the fake 50 NTD coins can be identified by the human vision when carefully inspecting the hidden letters in the anti-counterfeiting mark at a specific view angle. However, it is difficult to use this method in a vending machine. It was reported that the fake 50 NTD coin can be identified by using a scanning electron microscope with an energy dispersive X-ray analyzer [1]. It was also reported that, by using X-ray diffraction and X-ray fluorescence methods, multivariate cluster analysis can be applied to the identification of counterfeit coins [2]. A similar work reported that the distinction between authentic and counterfeit coins can be perceived by three-dimensional (3-D) video microscopy and environment scanning electron microscopy (ESEM) [3]. Such high-end techniques are high in accuracy, but it takes a lot of time to collect and process data. All these methods cannot be implemented for coin detection in automatic vending machines at an acceptably low cost. 

Another non-contact technology for detecting counterfeit coins is digital image processing. The pixels in the image of a coin are converted to the data array for comparison with the reference image to determine the cross-correlation in the two-dimensional (2-D) space [4]. However, the optical method suffers from the interference caused by the corrosion rusting and tarnishing on the surface of the coin. To enhance the accuracy of discrimination, the geometric information, including the height and depth of the text and the picture on the coin, is scanned to create a 3-D image and compared with each reference coin to identify counterfeit coins [5]. To advance this technology, neural network and deep learning to classify coins have been proposed [6]. Such methods require a computing-intensive system to automatically detect counterfeit coins, making it difficult to achieve a high throughput. 

To rapidly discriminate the coins in vending machines, various practical methods have been developed to recognize counterfeit coins with high throughput at a lower cost. The eddy current sensor is the dominant method used in common vending machines [7,8,9,10]. For example, Lu et al. [7] reported the method using two eddy current sensors operating at resonant frequencies to classify the coins based on the voltage distribution maps. Carlosena et al. [8] reported the improved multi-sensor technologies, including optical, electromagnetic, acoustic, and impact sensors, used to achieve good discrimination of coins for the high-end coin discriminator. The recorded acoustic signal is converted into the power spectrum to determine the coin’s vibration frequencies, and the results are compared with those for real coins. Gavrijaseva et al. [9] reported a similar acoustic method by detecting the sound of a Euro coin falling onto a metal plate. The sound was picked up by a microphone and the acoustic spectrum was post-analyzed using the fast Fourier transform (FFT) method. The coin classification method based on the detection of acoustic signals excited by the contact force can also be implemented with a higher throughput by using frequency counting. However, it is difficult to achieve accurate classification because of the unavoidable interference of the sound generated by the coin rolling and bumping in the track. Furthermore, the characteristic frequencies are also affected by the tapping position on the coin [10]. The non-ideal effects occurring in the contact testing method come from the fact that the magnitude and orientation of the contact impact force on a rolling coin are unstable.

To meet the requirement of rapid and reliable detection while lowering the system cost, it is necessary to devise a robust technique to classify counterfeits suitable for vending machines without physical contact with the coin. The electromagnetic acoustic transducer (EMAT) method is a solution that can produce sound on a coin and detect the acoustic signal, both in a non-contact manner. The EMAT method is widely applied in nondestructive testing such as thickness measurement, crack detection, and internal stress inspection in materials [11,12,13,14,15]. The most popular application is to identify hidden cracks. However, the low excitation efficiency and poor signal-to-noise ratio are the major hurdles for the applications of EMAT to the detection of small cracks [11]. Recently, several novel designs and excitation techniques are proposed to enhance the excitation efficiency of EMAT. For example, He et al. [11] presented the line-focusing EMAT with a meander-line coil to produce a chirp excitation signal. By employing the Barker-code pulse-compression technique, the allowed liftoff is increased, and the number of synchronous averages is reduced, which substantially improves the performance for rapid and high temperature application. Isla et al. [12] described the butterfly coils and cone-tipped magnets used in the EMAT to produce the shear acoustic waves with mono-polarity and high-mode purity. The size of the magnet core is investigated to optimize the normal component *B*_n_ of the flux density. Wang et al. [13] investigated the effectiveness of the magnets’ magnetic pole distribution while optimizing the amount of magnet elements placed around the tube sample. The torsional wave amplitude is increased by up to 60% when the poles in the magnet array are arranged antiparallel with the period matching the wavelength. Shi et al. [14] proposed the impedance matching method to improve the signal-to-noise ratio and range resolution for the spiral coil EMAT. The wire diameter of the spiral coil was explored in the range of 0.05 to 0.6 mm to enhance the pulse compression effect. The findings indicate that the pulse-compressed acoustic signal is optimized when the diameter is 0.15 mm and the matching parameters are appropriate, which allows an enhancement in the amplitude of the ultrasonic displacement signal by more than 3-fold, and a reduction in the width of the pulse envelope by 31.7%. Zhang et al. [15] designed a low-cost EMAT by combining printed-circuit-board (PCB) coils with two half-ring magnets. By comparing the geometrical dimensions of four distinct pairs of magnet half-rings, the signal amplitude of the shear-horizontal wave EMAT was optimized. The findings indicate that the signal amplitude increases as the inner diameter of the magnet reduces, reaching its maximum when the ratio of the wavelength to the outer magnet diameter is close to 1. To automate the material testing process and reduce the cost, microcontroller-based EMAT systems are also being developed. Cheng et al. [16] proposed the EMAT system consisting of an excitation circuit operating with a 600 V power supply and a signal reading circuit through an ADC converter. The excitation control and data transmission to the computer are performed by an 89S52 microcontroller. Zhong et al. proposed a series resonant EMAT system featuring good responses at the resonant frequency [17]. A microcontroller-based circuit comprising a full H-bridge of metal-oxide-semiconductor field-effect transistors (MOSFET) is used to generate the excitation current up to 60 A at the excitation voltage of 80 V. Another EMAT system with a microcontroller-based excitation generator is proposed by Bing et al. [18] using the microcontroller STC89C516 and the direct-digital-synthesis chip AD9850 to control the excitation signal. The circuit runs at a peak-to-peak voltage of 100 V and generates the excitation at the resonant frequency using a half-bridge MOSFET circuit. Despite the recent advancements in EMAT described above, all these works are intended for the nondestructive testing of plate [15], pipe [13], or bulk [14] materials. The design of EMAT for a microcontroller-based coin recognition system is not yet reported.

In this work, the coin classification system comprising an EMAT and a microphone receiver is proposed. A direct current (DC) voltage source powers the discharge capacitor circuit for generating the pulse current to drive the EMAT. The pulse current creates a transient magnetic field, which induces eddy currents in the coin and thereby produces an impact Lorentz force on the coin. The acoustic signals resulting from the impact are received by a microphone and filtered to extract the oscillating signal caused by the coin’s vibration. The signals are converted into square waves to determine the natural frequency of the coin’s vibration, and the observed frequency is used to determine the authenticity. The experimental and numerical results are analyzed and discussed. The performance of the prototype EMAT coin classification system is also demonstrated.

## 2. System Design and Working Principle

### 2.1. Experimental Coin Identification System

**Figure 1:**
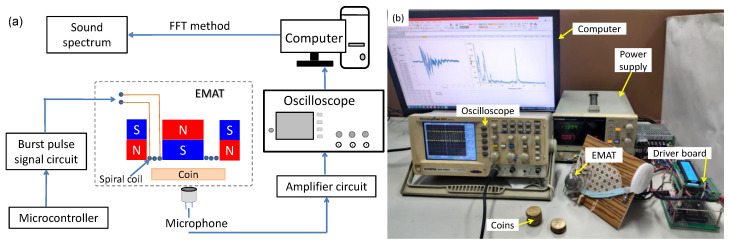
The experimental coin identification system based on an electromagnetic acoustic transducer (EMAT) and a microphone receiver: (**a**) functional diagram, (**b**) photograph of the system.

Figure 1a depicts the function block diagram of the experimental coin identification system based on an EMAT and a microphone receiver. The photograph of the system is shown in Figure 1b. When the coin approaches the spiral coil, it is hit by the Lorentz force induced by an eddy current pulse within it. The eddy current pulse interacts with the radial static magnetic field generated by a pair of concentric magnets. The magnet assembly with a spiral coil is a cylinder 38 mm in diameter and 20 mm in height. The high current pulse derived from the signal generated by a microcontroller is delivered by a discharge capacitor circuit to the spiral coil. A microphone near the bottom surface of the coin detects the vibration of the sound wave. In the experimental system, the detected acoustic signals are amplified and recorded by a digital storage oscilloscope at a sampling rate of 50 mega samples per second. The observed acoustic signal is converted into the frequency domain by the FFT method on the computer. The quality of the coin is assessed by the observed natural frequency received by the microphone.

### 2.2. Design of the EMAT for Coin Identification

**Figure 2:**
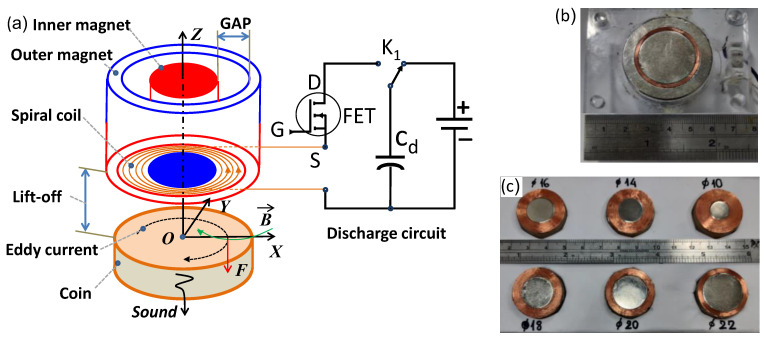
The prototype EMAT for coin identification: (**a**) working principle, the blue and red colors indicate the south and north magnetic poles; (**b**) the photograph of the magnet-and-coil assembly with a 25-mm inner magnet; (**c**) the photograph of the spiral-coil-and-inner-magnet sets with the diameter ranging from 10 mm to 22 mm.

The structure of the EMAT consisting of an outer ring magnet, an inner cylinder magnet, and a spiral coil is shown in Figure 2a. In all experiments, the outer magnet is an N45-grade ring-shaped NdFeB magnet, which is 27 mm in inner diameter and 38 mm in outer diameter. The inner magnet is an N35-grade cylindrical magnet with a diameter no greater than 25 mm. The photograph of the prototype EMAT probe with a 25-mm inner magnet is shown in Figure 2b. The inner magnet is kept concentric with the outer magnet by filling the gap with a tube-shaped plastic fixture made by a 3-D printing machine. The spiral coil, which induces the eddy current in the coin, is mounted on the top of the fixture. The spiral coil is driven by a discharge capacitor circuit whose excitation time is controlled through the G gate of the field-effect transistor (FET). The switch K1 is controlled by a microcontroller to change between the charging and discharging states, as shown in Figure 2a. The magnetic poles of the inner and outer magnets are aligned antiparallel to interlock each other. With this assembly, the magnetic field at the position above the gap between the inner and outer magnets is virtually parallel to the surface of the coin nearby. The interaction between the circumferential eddy current density $\vec{j}$ and the static magnetic field $\vec{B}$ will create an impulse Lorentz force perpendicular to the coin’s surface. The force density is calculated by [12]:

(1) $${\vec{f}}_{L}=\vec{j}\times \vec{B}$$

The non-contact impulse exerting on the charge carriers in the coin results in the deformation of the coin material, thereby inducing the acoustic wave emitted into the space in the vicinity of the coin. The coin vibrating inside the static magnetic field will induce the secondary eddy current oscillating at the same frequency as the acoustic wave on its surface [12]:

(2) $$\vec{J^{\prime }}=\sigma_{c}(\vec{v}\times \vec{B})$$

where $\vec{J^{\prime }}$ is the secondary eddy current density on the coin, *σ*_c_ is the electrical conductivity of the coin, $\vec{v}$ is the vibration velocity of the charged carrier in the coin, and $\vec{B}$ is the magnetic flux density. In principle, the secondary magnetic field produced by $\vec{J^{\prime }}$ can be picked up by the same excitation coil, but the signal level is low and sophisticated signal processing techniques are required to extract the interference-free signal. In our system, a microphone is used to receive the acoustic signal from the coin to simplify the circuit design. Since the EMAT and microphone are placed on the opposite sides of the coin, the force perpendicular to the surface of the coin is most effective to vibrate the coin in a surface-wave mode. Therefore, the strength in the radial component (*B*_r_) of the static magnetic field is of central importance to boost the sound signal. To optimize the magnitude of the sound, the outer diameter of the spiral coil filling the gap between the inner and outer magnets is 27 mm, which fits the inner diameter of the outer magnet and is just within the 28-mm diameter of the coin under test. The outer diameter of the inner magnet, which fits the inner diameter of the spiral coil, varies from 10 mm to 25 mm, while the gap size, i.e., the track width of the spiral coil, ranges from 8.5 mm to 1 mm, as shown in Figure 2b,c. The numbers of turns for the spiral coil and the diameters of magnets corresponding to various gap sizes are listed in Table 1.

**Figure 3:**
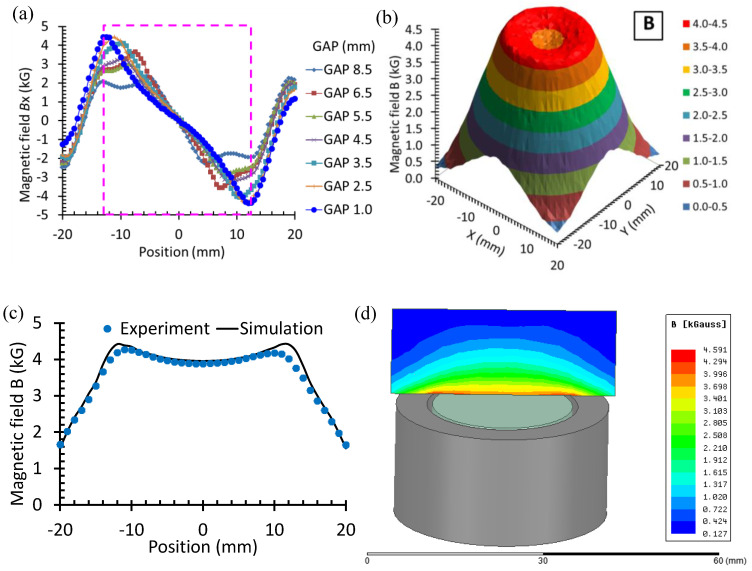
Magnetic field distribution at 2 mm above the magnet set of the EMAT: (**a**) The radial component *B_x_* along the *x*-axis for various gap sizes; (**b**) distribution of the total field *B* on the *x*–*y* plane for GAP = 1.0 mm; (**c**) the total field *B* along the *x*-axis obtained by measurement and simulation for GAP = 1.0 mm; and (**d**) simulation result of the total field on the central vertical plane above the magnetic set.

The distributions of the static field component *B_x_* along the *x*-axis for various assemblies of the magnet sets are shown in Figure 3a. The measured distribution of the total field magnitude for the magnet set with a 1-mm gap is shown in Figure 3b,c. The magnetic field components in the *x* = ±20 mm range are measured by a three-axis teslameter, model 3MTS from Senis, with the center of the Hall probe 2 mm above the surface of the magnet set. The field distribution is also calculated by numerical simulation using the ANSYS software. For the case with a 1-mm gap, the measured and simulated results are well in agreement with each other, as shown in Figure 3c. The maximum total field of 4.5 kG is achieved near the position of the gap. Figure 3d shows the simulated total-field distribution on the central vertical plane above the magnet set. It is found that the total field is maximized near the gap only when the liftoff distance is small. The above result shows that the maximum of the *B_x_* component increases up to 4.5 kG and dominates the total field as the gap decreases to 1 mm when the liftoff distance is 2 mm. The component *B_x_* is zero at *x* = 0 because the *B* vector is perpendicular to the surface of the magnet at the center, where *B* = *B*_z_. The maximum radial flux density is generally enhanced when reducing the gap. With a 1 mm gap, the maximum flux density occurs at *x* = ±12 mm, which is near the edge of the coin under test. In this case, the Lorentz force on the coin could be maximized, thereby enhancing the acoustic signal emitted from the vibrating coin.

### 2.3. Properties of the Coin

**Figure 4:**
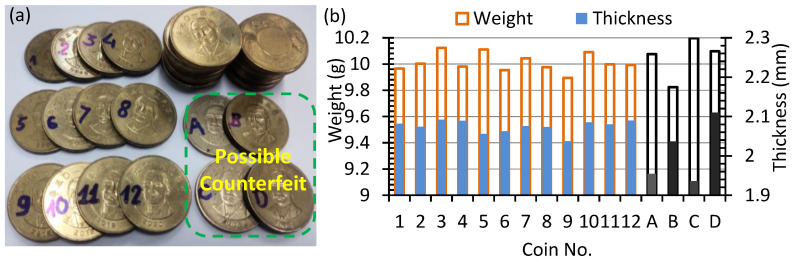
(**a**) The 50 NTD coin specimens used in this work; (**b**) weight and thickness distribution of the coin.

The 50 NTD coins, which have been circulating in Taiwan since 2002, are made of aluminum–copper alloy. Their mechanical properties and chemical composition are shown in Table 2. The specimen coins of this work are collected from retail stores, local markets, and vending machines in Taiwan in recent decades, and their authenticity is identified by inspecting the anti-counterfeiting mark. Twelve of the thirty authentic coins are marked with numbers from 1 to 12, and the four possible counterfeit coins without hidden letters are marked with A, B, C, and D, as shown in Figure 4a. The weight and average density are measured with a high precision scale with 1 mg resolution, and the thickness of the coin is measured using a digital micrometer with 1 μm resolution. The thickness is measured at the position away from the pattern and text minted on the surface of the coin. The average weight, density, radius, and thickness of the standard coins are shown in Table 2. The distributions of the weight and thickness for both authentic and possible counterfeit coins are shown in Figure 4b. It is found that the deviations from the standard weight of 10 g are 0.6%, −1.9%, 1.9%, and 0.9%, while the deviations from the standard thickness of 2.07 mm are −5.8%, −1.8%, −6.7%, and 1.7%, respectively, for the coins A, B, C, and D. For the coins A and C, the correlation between the weight and thickness is poor. Since the deviations from the standard radius of 14 mm are only −0.19%, −0.04%, −0.06%, and 0.10%, respectively, for the coins A, B, C, and D, the poor correlation between the weight and thickness implies that there exists significant variations in the mass density of the counterfeit coins. 

**Figure 5:**
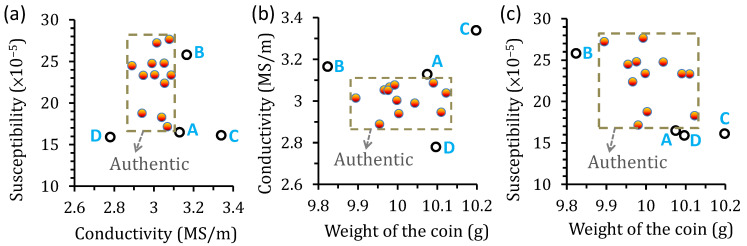
Electromagnetic properties and weight distribution of the 50 NTD coins: (**a**) susceptibility vs. conductivity, (**b**) conductivity vs. weight, and (**c**) susceptibility vs. weight.

The distribution of the observed weight and electromagnetic properties are shown in Figure 5. The electrical conductivity is measured by the four-probe method [19], and the susceptibility is obtained by the induction method using a solenoidal magnetizing coil and a pair of induction coils [20]. It is found that the weights of the real and possible fake coins A and D are indistinguishable, as shown in Figure 4b. The susceptibility of the possible fake coin B is also within the range of authentic coins, as shown in Figure 5a. In contrast, the conductivities of the counterfeit and real coins are dissimilar. Figure 5b shows the conductivity plotted with the weight, where the distinction between the cluster of real coins and the coins B and D is more apparent, but coin A is almost indistinguishable from the real ones. When the susceptibility is plotted with the weight, all the possible fake coins are distinguishable from the authentic coins, as shown in Figure 5c. These results indicate that it is less accurate to discriminate the coins simply based on their weight, conductivity, or susceptibility alone. By incorporating multiple characteristics, the accuracy of coin recognition can be significantly improved. 

The greater spread in the weights and electromagnetic properties of non-standard coins implies that there may be a significant deviation in the natural acoustic frequency of these coins, which can be estimated based on their mechanical properties. To estimate the fundamental natural frequency, the coin is assumed to be a circular plate of radius *r* and thickness *h*. The vibration mode is assumed to be an axially symmetric oscillation. When an elastic disk undergoes free edge vibration, the fundamental natural frequency is *f*_0,1_ of the mode without nodal diameter but one node circle, which can be estimated as follows [21]:

(3) $$f_{0,1}=\frac{\lambda_{0,1}^{2}}{2\pi r^{2}}\sqrt{\frac{D}{\rho h}}(\mathrm{Hz})$$

where *λ*_0,1_ is the dimensionless frequency parameter [21,22], *ρ* is the density, *h* is the thickness, *r* is the radius, *D = Eh*^3^/[12(1 − *ν*^2^)] is the flexural rigidity, *E* is Young’s modulus, and *ν* is the Poisson’s ratio. By using the parameters in Table 2 and the value of *λ*^2^ = 9.0031 reported in Ref. [22], the natural frequency predicted by (3) is 17.126 kHz for the standard coin. As the speed of acoustic wave in the coin is *v* = (*E*/*ρ*)^0.5^ = 3740 m/s, the acoustic wavelength at the natural frequency is more than 0.2 m, which is much larger than the thickness of the coin by two orders of magnitude. Therefore, the interference caused by the reverberation of the acoustic wave in the coin can be neglected.

The change in the natural frequency (Δ*f*_0,1_) of the coin by the variation in the geometric and mechanical properties can be derived from (3), as shown in Table 3. It is found that the relative change in natural frequency (Δ*f*_0,1_/*f*_0,1_) is more sensitive to the radius variation (Δ*r*) and thickness variation (Δ*h*). Nevertheless, the variation in radius is small and hence negligible for all coins. The frequency change is half the value of the variation in either elastic modulus (Δ*E*/*E*) or density (Δ*ρ*/*ρ*). According to the weight and thickness distribution in Figure 4b, the maximum density variation is Δ*ρ*/*ρ* = 1.8% for the authentic coins, which corresponds to the frequency change of 0.9% or 0.15 kHz, provided that the measured thickness is proportional to the average value on each coin. The variation in Poisson’s ratio (Δ*ν*) is least significant since the variation from *ν* = 0.3 by 1% results in the frequency change of only 0.099%. 

### 2.4. Optimal Excitation Pulse

The spiral coil of the EMAT probe is driven by the pulse current produced by a discharge circuit comprising a large capacitor, as shown in Figure 2a. The excitation pulse induces the eddy current and thereby the Lorentz force on the coin. The transient current pulse in the coil can be calculated from the parameters of the discharge circuit as follows:

(4) $$i(t)=\frac{U_{i}}{R_{d}(\lambda_{2}-\lambda_{1})}\left(\lambda_{2}e^{-\lambda_{1}t}+\lambda_{1}e^{-\lambda_{2}t}\right)\approx \frac{U_{i}}{R_{d}}\cdot e^{-\lambda_{1}t}$$

(5) $$\lambda_{1}=\frac{R_{d}}{2L}-\sqrt{\frac{R_{d}{}^{2}}{4L^{2}}-\frac{1}{LC_{d}}}\approx \frac{1}{R_{d}C_{d}}$$

(6) $$\lambda_{2}=\frac{R_{d}}{2L}+\sqrt{\frac{R_{d}{}^{2}}{4L^{2}}-\frac{1}{LC_{d}}}\approx \frac{R_{d}}{L}$$

where *i*(*t*) is the current as a function of time, *U_i_* = 5.4 V is the initial voltage of the capacitor, *R_d_* = 0.67 Ω is the total resistance of the discharge circuit, *C_d_* = 2.16 mF is the capacitance of the discharge capacitor, *L* = 2.84 μH is the inductance of the spiral coil, and *t* is the time. As *λ*_2_ ≫ *λ*_1_, the decay time of *i*(*t*) is dominated by the capacitive discharge behavior. 

**Figure 6:**
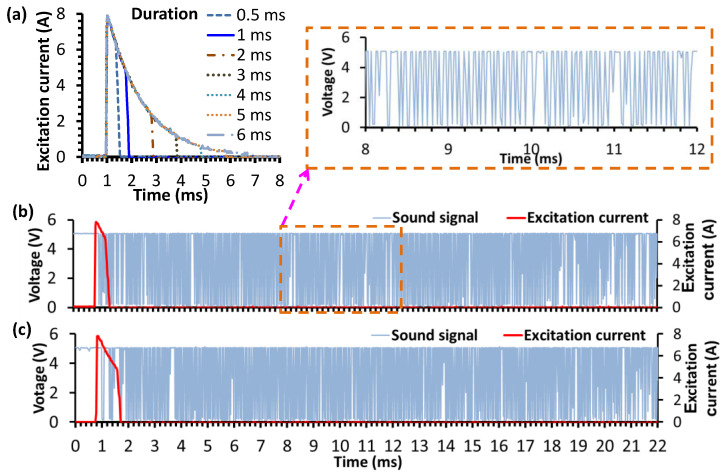
(**a**) The current pulse duration; the sound signal of coin No.1 after transforming the circuit with the different acting durations: (**b**) 0.5 ms, and (**c**) 1 ms.

The time duration of the excitation pulse was adjusted to explore its effect on the persistent time of the induced sound after the impact. The excitation duration is programmable by setting the on-time of the transistor using the microcontroller. Since the time for complete capacitive discharge is 6 ms, the excitation pulse is applied across the EMAT coil with a test duration ranging from 0.5 ms to 6 ms, and the audio signals after amplification are recorded, as shown in Figure 6a. It is found that the persistent time of the audio signals is quite similar for various excitation durations, suggesting that the decay time of the amplified sound signal depends weakly on the excitation duration longer than 0.5 ms. However, some oscillating cycles are missing with a 0.5 ms excitation period, as shown in Figure 6b,c. With an excitation period of 1 ms and above, the audio oscillation is stable and sustains for more than 20 ms, as shown in Figure 6c, which is sufficient for counting the natural frequency of the sound. The frequency counting method is conducted by recording the number of cycles of the square-wave-like signal processed by the transforming circuit, which is described in the following section. 

**Figure 7:**
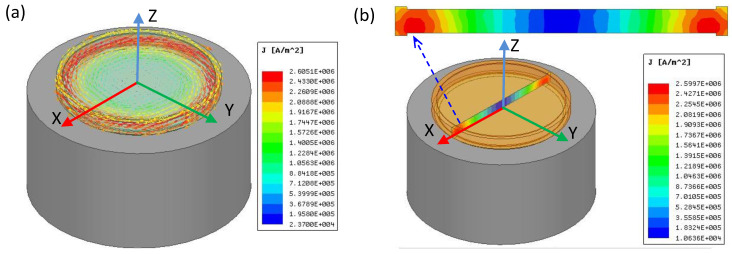
Simulated eddy current distribution in the coin at 0.5 ms after the excitation current pulse is applied: (**a**) current density vectors inside the coin; (**b**) intensity of current density on the central cross section of the coin. The horizontal range for the cross section is 28 mm.

Under the excitation current pulse, the eddy current is induced inside the coin immersed in the static magnetic field to generate the non-contact impulse force. To estimate the force on the coin, the distribution of eddy current density inside the coin is simulated using the finite element software ANSYS. Figure 7 shows the distribution of eddy currents at 0.5 ms after the 8-A excitation pulse is applied. It is found that the eddy current flows along the circumferential direction with the maximum intensity occurring near the edge, as shown in Figure 7a. The eddy current concentrates near the coin’s outer edge, where the radial component of the static magnetic field is also maximized. On the central cross-section, the maximum eddy current density is 2.6 A/mm^2^ at the position right above the spiral coil, as shown in Figure 7b. The total Lorentz force, which is estimated by integrating (1) over the volume of the coin, is 0.4 N perpendicular to the coin’s surface. As the impulse force concentrates on the rim of the coin, the coin will vibrate in a (0,1) mode, as described in (3).

### 2.5. Transforming Circuit for Sound Signal

**Figure 8:**
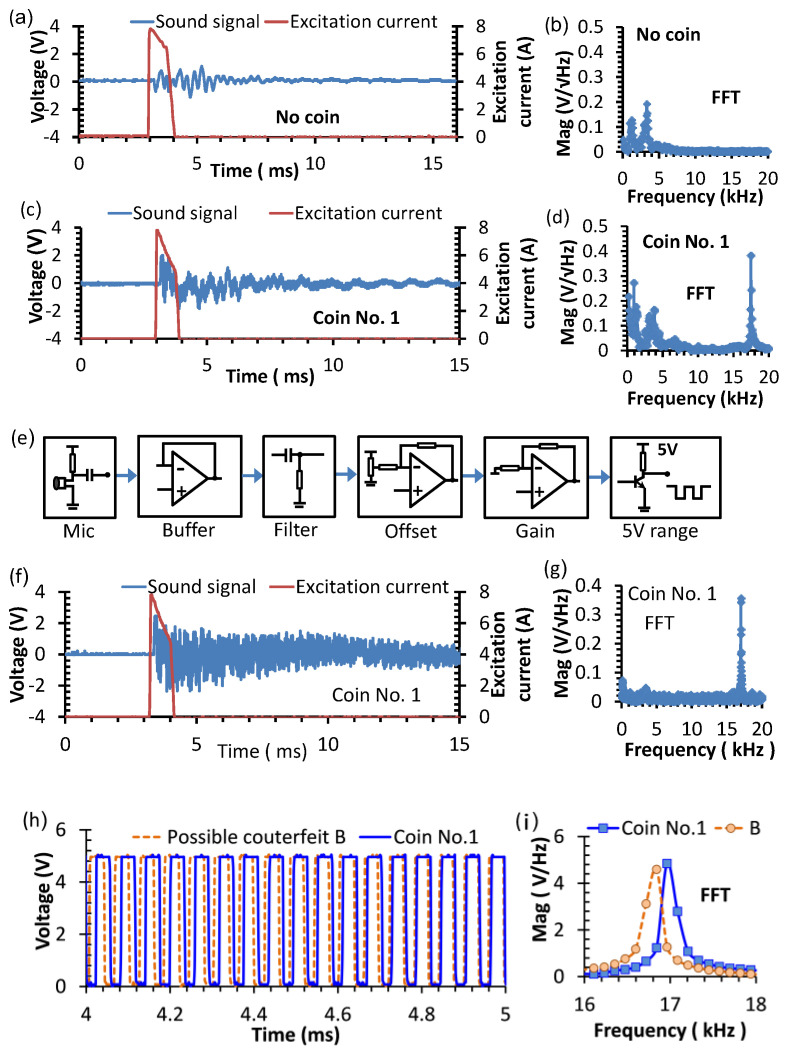
The original and transformed EMAT singals induced by a pulse current excitation: (**a**) the time-domain sound signal without a coin; (**b**) FFT spectrum of the signal without a coin; (**c**) the sound signal of the coin No.1 before filtering; (**d**) the FFT spectrum of the signal before filtering; (**e**) the functional diagram for the circuit transforming the sound signal to the square waveform; (**f**) the sound signal of the coin No.1 after filtering; (**g**) the FFT spectrum of the signal after filtering; (**h**) the sound signals of the coin No.1 and possible counterfeit coin B after passing the transforming circuit; and (**i**) the FFT spectrum of the signals after the transforming circuit.

With the experimental system, the background sound generated by the coil of the EMAT without a coin is recorded in the time domain by the oscilloscope, as shown in Figure 8a. The signals digitized by the oscilloscope are loaded into the computer for FFT processing. The FFT result shows that the background vibration frequency is less than 5 kHz, as shown in Figure 8b. When an authentic coin is excited with the EMAT probe to induce the acoustic signal, the characteristic frequency at around 17 kHz is observed, as shown in Figure 8c,d. As the background interference is more apparent before 6 ms, frequency counting can be performed well after 6 ms. Although the acoustic signal after 6 ms still contains the contributions from both the background (as in Figure 8a) and the coin vibration (as in Figure 8c), the frequencies of the background signal are lower than 5 kHz (as in Figure 8b,d), which can be suppressed by a high-pass filter circuit with a cut-off frequency of around 10 kHz. 

To improve the accuracy in resolving the natural frequency of coin vibration, a transforming circuit is proposed to convert the analog audio signal into an interference-free square-wave voltage signal. The functional flowchart for the transforming circuit is shown in Figure 8e. The sound signal received by the microphone is read by a buffer circuit to avoid the possible distortion arising from the circuit loading effect. Since the coin’s sound is induced by a strong excitation pulse, the sound received by the microphone must be processed to minimize the background electromagnetic interference. The background interference from the spiral coil is eliminated by a high-pass filter in the filter stage, of which the cutoff frequency is calculated by:

(7) $$f_{c}=\frac{1}{2\pi RC}$$

where *f_c_* is the cutoff frequency, *R* is the resistance, and *C* is the capacitance of the RC high-pass filter circuit. The signal after the filter stage contains a constant shift in DC level, which is removed by adjusting the potentiometer in the offset circuit. The signal is subsequently amplified by a non-inverting amplifier in the gain stage, for which the gain is set to make the output amplitude higher than the trigger point of the subsequent transistor circuit. Finally, the transistor comparator circuit transforms the signal into a 5-V square wave. The frequency spectrum of the coin’s sound signal after filtering is shown in Figure 8f. It is found that the background signal at frequencies lower than 5 kHz is eliminated by the filter in the transforming circuit, leaving only the characteristic natural frequency of the coin, as shown in Figure 8g. In comparison with the un-transformed signal in Figure 8d, the characteristic acoustic frequency of the coin is more distinct in the spectrum, indicating that the filtered signal is much more useful for coin identification.

The comparison in the square waves derived from the audio signals of the authentic coin No.1 and the possible counterfeit coin B after passing the transforming circuit are illustrated in Figure 8h. It is found that the transformed signal of coin B has a longer period than that of coin 1, indicating that the natural frequency of coin B is lower than that of a standard coin. The FFT spectrum in Figure 8i clearly shows the different peak frequencies of 16.8 kHz and 17.0 kHz, respectively, for coin B and coin 1.

In analyzing the frequency spectrum, the signal-to-noise ratio (SNR) of the spectral voltage amplitude is defined as the ratio of the peak value to the intrinsic noise density, which is 102 for the data in Figure 8b,d at 17 kHz. The frequency band of the major no-coin background signal is less than 5 kHz, which is outside the frequency range of interest. Therefore, the major background signal is filtered out by the transforming circuit and has a negligible effect on the frequency counting. 

Although the 1-ms pulse excitation current includes the multiple-frequency components, the maximum peak in the frequency spectrum occurs at less than 0.5 kHz, and the side-lobe amplitudes reduce by more than 30 dB at above 10 kHz, which are well below the fundamental natural frequency of 17 kHz for the coin. Their interference to the coin’s vibration occurs at the non-resonant frequency and ceases in less than 1 ms after the excitation duration. On the other hand, the resonant vibration at the natural frequency lasts more than 20 ms. The frequency counting starts 5 ms after the excitation duration, so the possible interference from the reverberation of fundamental and harmonics of the excitation currents are avoided. The remaining background signal is contributed by the Lorentz force interaction between the excitation coil and the magnet, in which frequencies lower than 10 kHz are eliminated by the high-pass filter in the transforming circuit. For the harmonics of the coin’s resonant vibration, the frequencies are well above 20 kHz, which exceeds the 20 kHz bandwidth of the microphone receiver. Therefore, harmonic interference of the resonant vibration is excluded by the bandwidth limit of the microphone and has a negligible effect on the accuracy of frequency counting.

## 3. Results and Discussion

### 3.1. Optimal Diameters for Coil and Magnets of EMAT Probe

**Figure 9:**
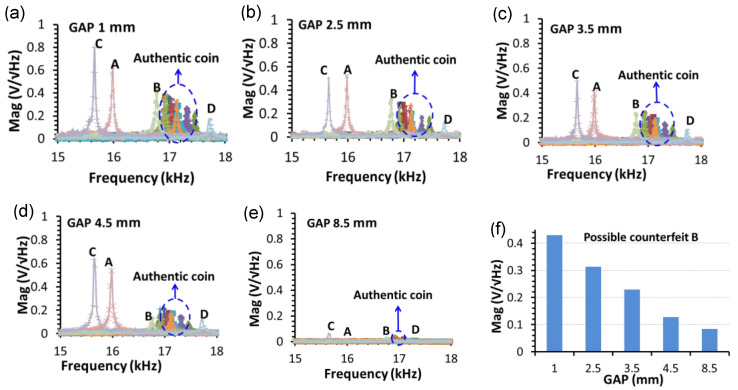
The natural frequency response of the coin with the different gap sizes of (**a**) 1, (**b**) 2.5, (**c**) 3.5, (**d**) 4.5, and (**e**) 8.5 mm. The corresponding diameters for the inner magnet are 25, 22, 20, 18, and 10 mm. (**f**) The magnitudes of the sound signal for the possible counterfeit B coin.

To determine the optimal diameters for the spiral coil and inner magnet sets of the EMAT probe, each gap case of the experimental system shown in Figure 2b,c is tested with the same excitation current on the same specimen coin. The EMAT signals for various gap values are recorded by a computer, then the FFT method is used to convert the sound signals from the time domain to the frequency domain, as shown in Figure 9a–e. The spectrum shows that every coin has a unique natural vibration frequency. The natural frequency of the authentic 50 NTD coins cluster in the range from 16.9 kHz to 17.45 kHz, while the possible counterfeit coins A, B, and C respond at lower frequencies of 15.9 kHz, 16.7 kHz, and 15.6 kHz, respectively, and the counterfeit D responds at a higher frequency of 17.73 kHz. An overview of all cases concludes that the EMAT method can be used for classification of the counterfeit and authentic coins. For the same coin, it is found that the amplitude of the natural vibration signal decreases monotonically with an increasing gap, as shown in Figure 9f. As the maximum amplitude is achieved with a 1-mm gap, the corresponding inner diameter of the spiral coil and the outer diameter for the inner magnet is 25 mm, which is used to build the coin identification system in the subsequent experiments. 

### 3.2. Optimal Liftoff Distance

**Figure 10:**
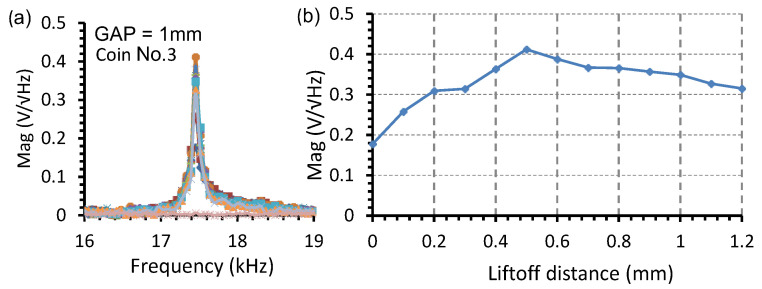
The natural frequency response of the coin No.3 tested with the EMAT with a 1 mm inter-magnet gap at the liftoff distance ranging from 0 to 1.2 mm: (**a**) the frequency spectra; (**b**) the spectral magnitude of the output voltage.

To maximize output sensitivity, the natural frequency of the sound is recorded at different liftoff distances, which are defined as the spacing between the spiral coil and the coin under test. Figure 10 shows the natural frequency of coin No.3 tested with the EMAT with a 1 mm inter-magnet gap at a liftoff distance ranging from 0 to 1.2 mm. The frequency spectra were recorded at various liftoff distances in a 0.1 mm step using the equipment setup in Figure 1. The excitation pulse in the coil of EMAT has a peak amplitude of 8 A and a duration of 1 ms. The coin No. 3 is randomly chosen to represent the standard coin for investigating the optimal liftoff distance to enhance the acoustic signal excited by the Lorentz force. The position of the coin is kept constant with its center coinciding with the central Z-axis of the excitation coil. The liftoff distance is defined by inserting one or several pieces of 0.1 mm thick plastic spacers. The sound signals from the coin detected by the microphone receiver are recorded by a digital storage oscilloscope. The data are subsequently analyzed using a computer software to obtain the FFT spectra, as shown in Figure 10a. The FFT spectra show that the natural frequency is constant at 17.27 kHz, despite the varying liftoff distance. As shown in Figure 10b, the optimal liftoff distance is found to be 0.5 mm, which maximizes the spectral magnitude to 0.41 V√Hz at the natural frequency.

### 3.3. Coin Classification Using a Microcontroller-Based EMAT System

**Figure 11:**
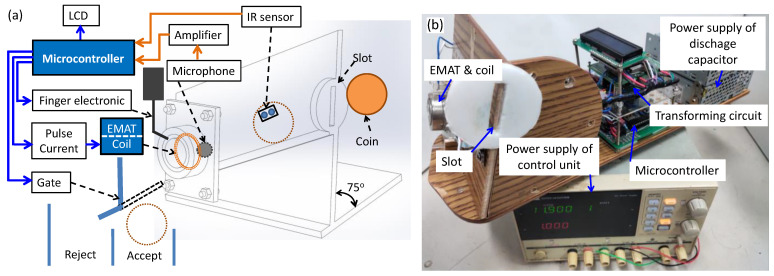
The microcontroller-based coin sorter with EMAT for classification of counterfeit and real coins: (**a**) functional diagram; (**b**) photograph of prototype.

**Figure 12:**
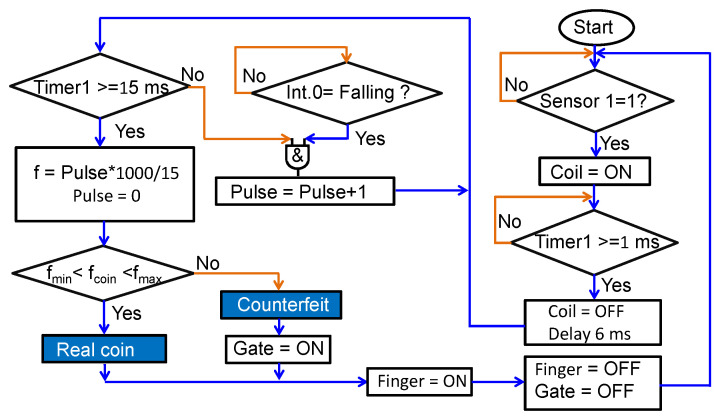
Algorithm of the firmware code in the microcontroller for frequency counting and coin classification.

This study aimed at building a stand-alone coin classification system based on the natural acoustic frequency induced by the contactless impact generated by an EMAT probe without the use of an oscilloscope and a computer. Since the FFT algorithm requires large memory and high computation power, it is difficult to implement the method on a microcontroller with a low-clock speed. A more feasible method to determine the frequency is to count the voltage pulses processed by the transforming circuit in a given duration by using the built-in counter and clock functions of the microcontroller. In our microcontroller-based coin identification system, the central processing unit is ATmega 328 with a 1K-byte electrically erasable programmable read-only memory (EEPROM) operated at a 16 MHz clock. The square-wave signal similar to those in Figure 8f is used to analyze the frequency of coin vibration. The frequency of the coin’s sound is counted by a microcontroller, with which the technique can be easily applied to vending machines. The functional diagram of the microcontroller-based coin sorter with EMAT is shown in Figure 11a, and the photograph is shown in Figure 11b. The algorithm of the firmware code for frequency counting and coin classification is shown in Figure 12.

Firstly, the coin is inserted into the small slot on the top of the track and then rolls down to the end of the track where an electronic finger will hold it. The track is tilted at an angle of 75° to avoid the liftoff distance variation during sliding. An infrared (IR) proximity sensor is mounted in the middle of the track to trigger the process for coin identification. When a coin passes by the IR sensor, the output of the IR sensor becomes high and triggers the discharge capacitor circuit to deliver a pulse current of 1-ms duration into the spiral coil. The current pulse induces the eddy current in the coin, thereby inducing the impact Lorentz force which exerts simultaneously on both the coil and the coin. The impact on the coin produces an acoustic sound to be received by the microphone placed on the opposite surface of the coin. The received acoustic signal is converted into a square wave with the transforming circuit shown in Figure 8e. A 6-ms delay timer is set to avoid the reception of unstable data at the beginning of the transient audio signal. Then, Timer1 is set to 15 ms and assigned to interrupt 0 (int.0) of the microcontroller for counting the number of pulses in this duration. The frequency is obtained by calculating the number of pulses of the audio signal per second. The frequency value is shown on a liquid crystal display (LCD) and compared with minimum and maximum values to determine the coin type. Finally, the gate at the end of the track is controlled by the obtained result to decide whether to accept or reject the coin. The minimum time for the coin to move from the slot to the gate is 0.4 s, which is much longer than the 22 ms time for determining the authenticity using the EMAT method. The duration of 0.4 s is acceptable for application in a vending machine since it is set by the time required for the movement of the coin through the coin sorter.

**Figure 13:**
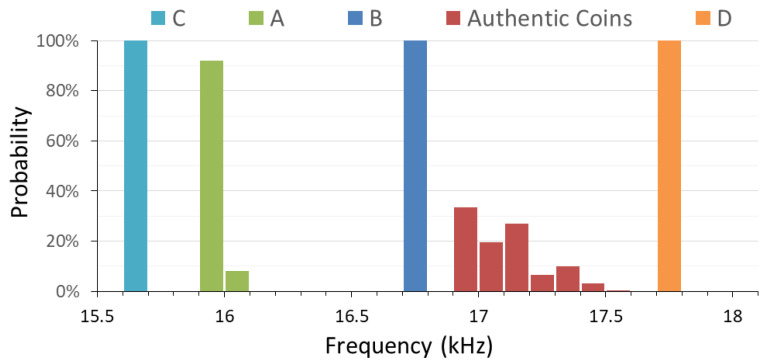
The classification result of the authentic and possible counterfeit coins tested by the proposed EMAT coin identification system with a microcontroller.

To evaluate the performance of the microcontroller-based coin classification system, thirty authentic and four possible counterfeit 50 NTD coins shown in Figure 4 were identified by using the EMAT method and repeated 100 times for every coin; the results are shown in Figure 13. It can be seen that the natural frequencies of the authentic coins form a cluster group ranging from 16.92 kHz to 17.45 kHz, while the counterfeits locate out of this group. The frequency of coin D is higher, and those of the coins A, B, and C are lower. The average natural frequencies are, respectively, 16.02, 16.73, 15.62, and 17.71 kHz for the possible counterfeit coins A, B, C, and D. The standard deviations are, respectively, 18, 19, 11, and 13 Hz. For all the coins, the average standard deviation (ASD) in natural frequency determined by the microcontroller-based EMAT system is δ_f_ = 12 Hz, which is less than 0.1% of the natural frequencies and much lower than the minimum difference (MD) of 192 Hz between the average natural frequencies of the authentic and possible fake coins. For frequency counting, the SNR is represented by the ratio of the natural frequency to the ASD. With the observed ASD of 0.012 kHz, the corresponding SNR is 1425. The minimum SNR (≈255) is the product of the natural frequency (≈17 kHz) and the available number of counts in the time duration (≈15 ms) for frequency counting.

According to Figure 4b, the variations in thickness from the average value of the standard coin are −5.8%, −1.8%, −6.7%, and 1.7%, and the mass differences from the standard value of 10 g are 0.7%, −1.8%, 2.0%, and 1.0%, respectively, for coins A, B, C, and D. The observed differences from the standard natural frequency of 17.1 kHz are, respectively, −6.7%, −2.1%, −8.6%, and 3.6%, which are generally correlated with the thickness variation. Since the variation in radius is small, the other factor contributing to the discrepancy in the variations between natural frequency and thickness is most likely the variation in elastic modulus.

**Figure 14:**
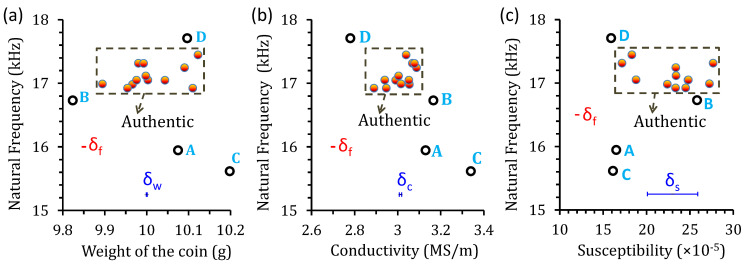
Distribution of the average natural frequency with respect to the (**a**) weight, (**b**) conductivity, and (**c**) susceptibility. The horizontal error bars (blue) indicate the ASDs for the weight, conductivity, and susceptibility, while the small vertical error bars (red) are the ASD for the natural frequency.

When the observed natural frequency is plotted with the weight, conductivity, and susceptibility, the distinction between the cluster of real coins and the possible fake coins are more apparent, as shown in Figure 14a–c. The corresponding ASDs for all coins and the MDs for the possible fake coins are listed in Table 4. It can be seen that the natural frequency is the most distinct feature since the minimum difference between the coin B and the closest authentic coin is 16 times the ASD, which is well sufficient to accurately discriminate the authenticity and is better than other features, i.e., permeability, conductivity, and weight. For coin recognition, the minimum SNR can be defined as the ratio of the minimum difference (MD) to ASD, i.e., MD/ASD, for the feature to be extracted. The minimum SNR is 16 with the natural-frequency feature, which is much better than the SNRs of all other features illustrated in Table 4. 

The performance of the proposed EMAT coin identification system can be further demonstrated by comparison with the existing acoustic method reported in the literature, as shown in Table 5. For the contact-force method reported in [10], the inaccuracy in determining the first-peak acoustic frequency of the coin ranges from 0.4% to 0.5%, corresponding to the SNR between 250 and 200. For our EMAT system, the inaccuracy is only 0.07% and the SNR is 1425, which is 6 to 7 times better than the contact-force method. These results demonstrate that the microcontroller-based EMAT system can improve the accuracy in coin recognition by hitting the coin with a non-contact force and counting the acoustic vibration frequency in less than 30 ms. 

The eddy current sensor is the predominant non-contact method used in common vending machines [8]. The working principle is to detect and analyze the signal caused by the coil’s impedance change, which is related to the conductivity and susceptibility, to determine the coin’s authenticity. However, the impedance is sensitive to the liftoff effect and hence cannot be determined with high accuracy in real-time in a non-contact manner. In contrast, the natural frequency is insensitive to the liftoff effect and can be accurately determined without contact in less than 30 ms using our prototype EMAT coin discriminator. As the proposed method does not require Fourier transform calculation, very little computation time and resources are needed. The transforming circuit converts the audio signal into a square wave in real-time, so the time required for the identification of the coin is determined by the excitation duration of the EMAT signal and the time needed for frequency counting, for which the overall processing time of 22 ms can be achieved, much less than the total time of 0.4 s for the coin to pass through the coin discriminator. The results show that the proposed EMAT method is an excellent solution for excluding counterfeit coins with a microcontroller-based system, which is valuable for building an accurate and rapid coin discriminator with multiple sensors at a low cost. The proposed technique is particularly attractive for use in common vending machines.

## 4. Conclusions

We have built a microcontroller-based EMAT system for coin identification and tested its performance in analyzing the natural acoustic frequency of a coin. The acoustic response induced by the EMAT is converted to a square wave signal by a transforming circuit. The natural acoustic frequency is determined in 22 ms by a microcontroller after the non-contact impact force is imposed. The prototype EMAT system achieves the highest response at the 0.5 mm liftoff distance when testing the 50 NTD coin. The natural acoustic frequency is accurately determined and consistent with the value predicted by the analytic calculation for coin vibration. The theoretical and experimental analysis shows that the change in natural frequency of the possible counterfeit 50 NTD coins are most likely dominated by the variation in their thickness and elastic modulus. The proposed non-contact acoustic method can detect the natural frequency of a coin with a signal-to-noise ratio of 1425, which is significantly better than the contact-force acoustic method for coin recognition reported to date. By using the proposed system, the obtained information is sufficient to confirm the authenticity of the coin, and the accuracy can be further enhanced by employing the detection method based on the other features. The proposed method has the potential to significantly improve the accuracy of the coin discriminator in automatic vending machines and ticket machines.

## Figures and Tables

**Table 1:** The dimensions of magnets and the number turns of the coil.

Gap (mm)	Diameter of Inner Magnet (mm)	Spiral Coil (Turn)
1	25	7
2.5	22	13
3.5	20	17
4.5	18	21
5.5	16	25
8.5	10	35

**Table 2:** Technical parameters for the 50 NTD coin.

Symbol	Material/Property	Parameter
	Copper alloy	Cu 92%, Al 6%, Ni 2%
*E*	Young’s modulus	110 GPa
*ρ*	Density	7.865 g/cm^3^
*h*	Thickness	2.07 mm
*r*	Radius	14 mm
*ν*	Poisson’s ratio	0.3

**Table 3:** Change in the natural frequency *f*_0,1_ by the variation in the geometric and mechanical properties of the coin.

Properties	Variation	Δ*f*_0,1_/*f*_0,1_	Δ*f*_0,1_/*f*_0,1_ for 1% Variation
Radius, *r*	Δ*r*	−2Δ*r*/*r*	−2%
Thickness, *h*	Δ*h*	Δ*h*/*h*	1%
Elastic modulus, *E*	Δ*E*	0.5Δ*E*/*E*	0.5%
Density, *ρ*	Δ*ρ*	−0.5Δ*ρ*/*ρ*	−0.5%
Poisson’s ratio, *ν*	Δ*ν*	*ν*Δ*ν*/(1 – *ν*^2^)	0.099% (*ν* = 0.3)

**Table 4:** Comparison between the minimum differences (MD) of permeability, electrical conductivity, weight, and natural frequency in terms of average standard deviations (ASD).

Features	ASD for All Coins	MD/ASD for Counterfeit Coins			
		A	B	C	D
Magnetic Susceptibility	δ_s_ = 5.79 × 10^−5^	0.11	0	0.18	0.22
Electrical Conductivity	δ_c_ = 0.0109 MS/m	3.8	7.1	23.1	10
Weight	δ_w_ = 0.00215 g	0	33	35	0
Natural Frequency *	δ_f_ = 0.012 kHz	81	16	109	22

* Real-time measurement.

**Table 5:** Comparison in performance between the contact force [10] and EMAT methods.

Method	Natural Frequency (kHz)	ASD (% of Natural Frequency)
Contact force	7.36−7.40 *	0.4−0.5%
EMAT	16.92−17.45	0.07%

* First-peak frequencies listed in Table 1 of Ref. [10].

## Data Availability

Not applicable.
